# Lysozyme Inhibitors as Tools for Lysozyme Profiling: Identification and Antibacterial Function of Lysozymes in the Hemolymph of the Blue Mussel

**DOI:** 10.3390/molecules28207071

**Published:** 2023-10-13

**Authors:** Lise Vanderkelen, Joris M. Van Herreweghe, Chris W. Michiels

**Affiliations:** Leuven Food Science and Nutrition Research Centre (LFoRCe), Lab Food Microbiology, Department Microbial and Molecular Systems, KU Leuven, B-3001 Leuven, Belgium; lise.vanderkelen@gmail.com (L.V.); joris.van.herreweghe@telenet.be (J.M.V.H.)

**Keywords:** lysozyme, lysozyme inhibitor, innate immunity, antibacterial activity, bivalves

## Abstract

Lysozymes are universal components of the innate immune system of animals that kill bacteria by hydrolyzing their main cell wall polymer, peptidoglycan. Three main families of lysozyme have been identified, designated as chicken (c)-, goose (g)- and invertebrate (i)-type. In response, bacteria have evolved specific protein inhibitors against each of the three lysozyme families. In this study, we developed a serial array of three affinity matrices functionalized with a c-, g-, and i-type inhibitors for lysozyme typing, i.e., to detect and differentiate lysozymes in fluids or extracts from animals. The tool was validated on the blue mussel (*Mytilus edulis*), whose genome carries multiple putative i-, g-, and c-type lysozyme genes. Hemolymph plasma of the animals was found to contain both i- and g-type, but not c-type lysozyme. Furthermore, hemolymph survival of *Aeromonas hydrophila* and *E. coli* strains lacking or overproducing the i- type or g-type lysozyme inhibitor, respectively, was analyzed to study the role of the two lysozymes in innate immunity. The results demonstrated an active role for the g-type lysozyme in the innate immunity of the blue mussel, but failed to show a contribution by the i-type lysozyme. Lysozyme profiling using inhibitor-based affinity chromatography will be a useful novel tool for studying animal innate immunity.

## 1. Introduction

Lysozymes (EC 3.2.1.17) are a heterogeneous family of enzymes that specifically hydrolyze the β-1,4 glycosidic bond between N-acetyl muramic acid and N-acetyl glucosamine in the bacterial cell wall polymer peptidoglycan, and thereby induce cell lysis. As such, lysozymes have been studied as alternative antimicrobials in a wide range of fields, including medicine, veterinary medicine, food, feed, and crop protection [1]. Based on differences in amino acid sequence, biochemical and enzymatic properties, lysozymes are classified into several types. In animals, three major types have been described: the c-type (chicken or conventional type, glycoside hydrolase (GH) family 22), the g-type (goose type, GH 23), and the i-type (invertebrate type, GH 22). These lysozymes have a different distribution, and animals can produce only one, two, or three different types. In addition, they commonly have two or more paralogs of each type. For example, vertebrates have c- and g-type lysozymes, insects have c- and i-type lysozymes, nematodes produce only the i-type lysozyme, and molluscs produce all three types [1,2]. Despite a low overall amino acid sequence identity, c-, g- and i-type lysozymes are believed to stem from a single ancestor and to have diversified after gene duplication events [2,3]. While the primary function of lysozyme in animals was probably related to antibacterial defense, this diversification has created novel functions such as facilitating the digestion of bacterial biomass as a source of nutrients. Several c-, g- and i-type lysozymes in various animals have been proposed to have a digestive function based on their expression in the gut, their acidic pH optimum (for lysozymes active in the stomach), and their resistance to digestive proteases. This function has been particularly well established in ruminants, where these lysozymes are highly expressed in the “true” stomach compartment (i.e., the acidic abomasum), and help to digest the microbial biomass that arrives from the upstream “fermentor” compartment (i.e., the nonacidic rumen) [1,2]. A novel role that was recently proposed is that lysozymes may help in the establishment of healthy microbiomes by providing a competition benefit to protective bacteria compared to pathogens. This was shown in the worm *Caenorhabditis elegans*, where *Enterococcus faecalis* induces the expression of a host lysozyme that limits growth of the pathogen *Staphylococcus aureus*, but to which *E. faecalis* itself is not very sensitive. The authors concluded from these findings that *E. faecalis* benefits from a competitive advantage due to this host response [4]. The individual contribution of different lysozymes in an organism is only beginning to be understood, based on the analysis of the spatial and temporal expression patterns of the lysozyme genes and their response to bacterial challenge, and by making use of gene silencing or knock-out techniques [3,5]. Transgenic animals (over-) expressing heterologous lysozymes have been reported to have increased pathogen resistance, which is of particular interest in the field of aquaculture where bacterial disease is a major problem [6].

While methods like analysis of gene expression and gene silencing and knockout are very powerful today, they also have some limitations in studying the function of lysozymes. Methodologically, they require knowledge of the genome sequence or at least of the lysozyme genes of the organism of interest and, for gene silencing and knockout, also a toolbox for genetic transformation and laboratory culture. For many animals, one or both of these are still missing. Furthermore, gene expression studies are mostly conducted at the transcriptional level and therefore do not necessarily reflect the quantity and activity of the enzymes. A method allowing the isolation and/or identification of different types of lysozymes would therefore be a useful complementary tool to study lysozyme activity and antibacterial properties. To this end, in the present work we explore the use of highly specific bacterial lysozyme-binding proteins.

To defend themselves against the bacteriolytic action of lysozymes, bacteria have evolved highly specific proteinaceous lysozyme inhibitors. The first such inhibitor was discovered in *Escherichia coli* in 2001 and was named Ivy (inhibitor of vertebrate lysozyme) [7]. Later, several additional inhibitors have been identified in different bacteria, with specificity against c-type [8,9], g-type [10], and i-type lysozymes [11], and there is increasing evidence that these inhibitors contribute to bacterial symbiosis or pathogenesis by allowing evasion of their host’s immune defense [12,13,14,15,16]. Several of these inhibitors have been well characterized and show a very high affinity for their cognate lysozymes. In the present work, we propose the use of such lysozyme inhibitors to detect c-, g-, and i-type lysozymes in biological samples and isolate them. Besides Ivy, we used PliG (periplasmic lysozyme inhibitor of goose-type lysozyme) and PliI (periplasmic lysozyme inhibitor of *i*nvertebrate lysozyme). These inhibitors have binding constants of 10^9^ M (Ivy), 5 × 10^8^ M (PliG), and 2 × 10^10^ M (PliI) [7,10,11]. We constructed a serial array of three affinity chromatography columns containing resins to which these inhibitors are covalently bound and demonstrated that it allows the one-step separation of c-, g-, and i-type lysozymes from complex mixtures. Using this array, which we named a Lys-Trap, we demonstrate for the first time that the hemolymph of the blue mussel not only contains i-type but also g-type lysozyme, and we provide evidence that the latter plays a role in antibacterial defense. 

## 2. Results and Discussion

### 2.1. Validation of c-Lys-, g-Lys-, and i-Lys-Trap Columns for Lysozyme Profiling

The c-type (Ivy), g-type (PliG), and i-type (PliI) inhibitors were recombinantly produced in *E. coli*, purified, and subsequently covalently coupled to a Sepharose resin. The functionalized resins were then used to fill three columns for conducting affinity chromatography. Each column was first tested separately by loading it with 0.125 to 1.0 mg of the corresponding lysozyme, i.e., HEWL, SalG, and VpL. Even with the maximal amount of applied lysozyme, no residual enzyme activity was observed in the fractions collected from the end of the columns during the loading and washing step, which are hereafter referred to as the flow-through fractions. This indicated that the lysozymes were efficiently bound to the columns. Moreover, after elution with a linear gradient of salt and alkaline pH, the lysozymes were recovered without apparent loss in the amount loaded and in the enzymatic activity in the *M. luteus* turbidity assay (data not shown; only a single validation experiment conducted). 

**Figure 1:**
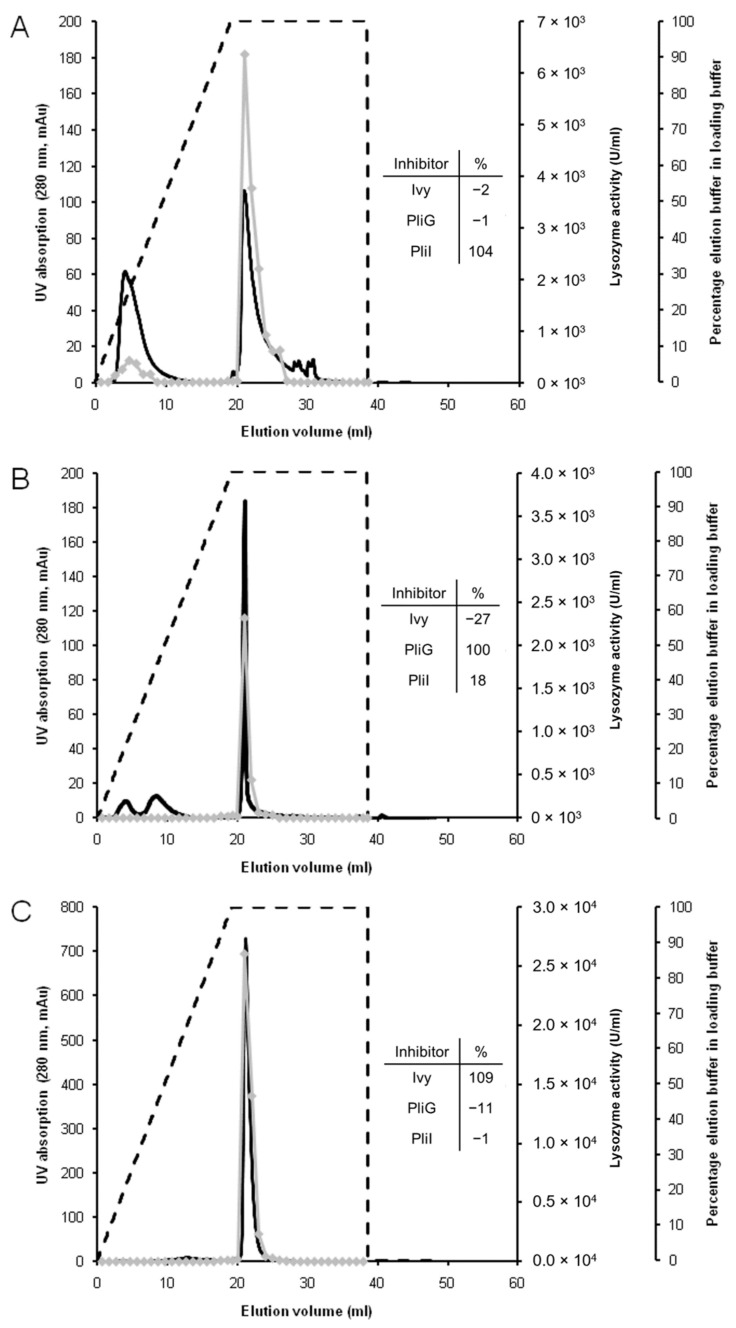
Validation of the serial Lys-Trap with a three-lysozyme mixture. A serial array of affinity columns functionalized with the lysozyme inhibitors PliI, PliG, and Ivy, respectively, was loaded with a mixture of three corresponding lysozymes (VpL, SalG, and HEWL), and each column was subsequently separately eluted. The panels represent the elution profiles of the PliI column (**A**), the PliG column (**B**) and the Ivy column (**C**). Dashed line: elution gradient, i.e., the percentage of elution buffer 0.1 M (Tris-HCl pH 12.0 + 2 M KCl) in the loading buffer (0.1 M Tris-HCl pH 7.0). Black line: UV absorption at 280 nm expressed as milli-absorption units (mAU). Grey line with filled triangles: lysozyme activity in the fractions expressed as IU/mL. The inset tables show the % inhibition of the lysozyme activity in the pooled peak fractions by the three lysozyme inhibitors.

In a subsequent experiment, a serial array of the three columns (i-, g-, and c-type, respectively) was tested for its ability to separate a mixture of 1 mg of each VpL, SalG, and HEWL in a single run. After loading of the mixture, each column was eluted separately and produced a major protein peak with lysozyme activity near the end of the gradient (Figure 1). As indicated in the inset tables in Figure 1, these peak fractions from each Lys-Trap were inhibited specifically by their corresponding inhibitor and not by the other inhibitors, thus confirming the identity and purity of the eluted proteins. The i-Lys-Trap also produced a minor peak with some lysozyme activity at the beginning of the elution gradient (Figure 1A). This activity was partly inhibited by Ivy (69%), PliI (14%), and PliG (1.5%). Therefore, this peak is probably the result of non-specific binding of the three lysozymes onto the first column of the array. The inclusion of a low amount of salt in the binding buffer may possibly prevent this non-specific binding in future analyses. In conclusion, this validation experiment confirms the ability of the lysozyme trap array to separate mixtures of c-, g-, and i-type lysozymes.

### 2.2. Lysozyme Profiling of Mussel Hemolymph Plasma

**Figure 2:**
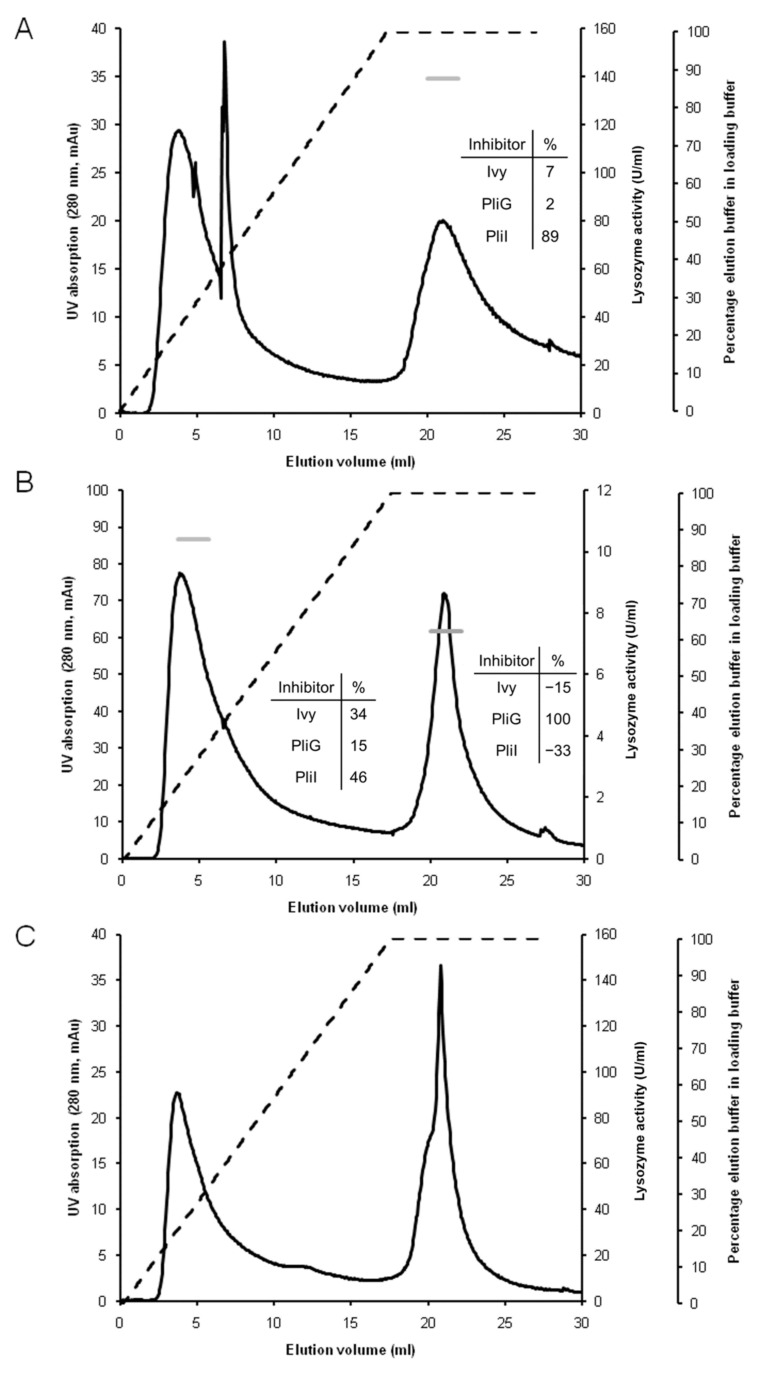
Lysozyme profiling of blue mussel hemolymph plasma using the serial Lys-Trap. A serial array of affinity columns functionalized with the lysozyme inhibitors PliI, PliG, and Ivy, respectively, was loaded with blue mussel hemolymph plasma, and each column was subsequently separately eluted. The panels represent the elution profiles of the PliI column (**A**), the PliG column (**B**), and the Ivy column (**C**). Dashed line: elution gradient, i.e., the percentage of elution buffer (0.1 M Tris-HCl pH 12.0 + 2 M KCl) in the loading buffer (0.1 M Tris-HCl pH 7.0). Black line: UV absorption at 280 nm expressed as milli-absorption units (mAU). Horizontal grey stripes: lysozyme activity in the pooled peak fractions expressed as IU/mL. The inset tables show the % inhibition of the lysozyme activity in the pooled peak fractions by the three lysozyme inhibitors.

The hemolymph of the blue mussel was chosen to validate the Lys-Trap array for lysozyme profiling of biological samples for three main reasons. First, fresh animals can be easily purchased since it is a popular seafood. Second, like other mollusks, the species has genes for all three types of lysozyme [2,17]. Third, although the hemolymph of the blue mussel is known to contain i-type lysozymes [18], the presence of g- and c-type lysozymes has not yet been demonstrated. Together, these elements make the blue mussel an ideal testcase to validate the usefulness of the Lys-Trap array. After loading 100 mL of hemolymph plasma onto the array and washing away unbound proteins, the three columns were eluted separately with a combined linear salt and pH gradient. Elution of the c-Lys-Trap column (last in the array) resulted in two UV absorption peaks, one at the start and one towards the end of the elution gradient, but neither of them showed lysozyme activity (Figure 2C). This most likely means that there is no c-type lysozyme present in the hemolymph, although we cannot formally exclude the existence of a c-type lysozyme that is not inhibited by Ivy. Protein blast analysis of *M. edulis* genome sequences in Genbank with HEWL yielded a single putative c-type lysozyme gene, compared to four in the close relative *M. galloprovincialis* (Supplementary Figure S1). Only one of these four has been studied in detail, and found to be transcribed in multiple tissues and to be induced in hemocytes by bacterial challenge, suggesting a role in innate immunity [19]. 

Elution of the g-Lys-Trap (second in the array) produced two UV absorption peaks, both showing lytic activity (10 and 7.4 U/mL, respectively) (Figure 2B). The lytic activity of peak 1 could not be unequivocally assigned to any of the three lysozyme types since it was partly inhibited by all three inhibitors. Since the early elution implies weak and possibly aspecific binding, and since our experience is that low levels of inhibition in the *M. luteus* lysozyme activity are not always reliable, no further attention was given to this peak. Peak 2, in contrast, clearly corresponds to a g-type lysozyme based on its inhibition profile (see inset of Figure 2B). Bivalves generally contain several gene alleles of putative g-type lysozymes and some studies have addressed the differential expression of the alleles and their induction by bacterial challenge, and even the localization of the proteins by immunofluorescence microscopy. In *M. galloprovincialis*, the expression and enzyme properties of two (recombinant) g-type lysozymes was studied. MGgLYZ1 had maximal activity at pH 6 and was induced in hemocytes by bacterial challenge, implying an immune-related function. MGgLYZ2, on the other hand had a pH optimum of 4–5 and had less protease cleavage sites, possibly reflecting a digestive function [20]. A recent study reported that transcription of a g-type lysozyme gene was constitutive in the digestive gland and upregulated in the hemocytes and gills of the scallop *Agropecten purpuratus*. Immunofluorescence microscopy analysis confirmed the presence and induction of the lysozyme in hemocytes. Furthermore, transcript silencing by RNAi caused increased levels and a compositional shift of the hemolymph microbiome, implicating the g-type lysozyme in immune defense or modulation [21]. Our present data prove the presence of a g-type lysozyme in the hemolymph plasma of *M. edulis*. Blast analysis of whole genome sequences in Genbank with salmon g-type lysozyme reveals three putative g-type lysozyme genes in *M. edulis*, compared to four in *M. galloprovincialis* (Supplementary Figure S2). Further analysis by mass spectrometry could clarify which of the three are present in the hemolymph plasma. 

Finally, elution of the i-Lys-Trap generated three UV absorption peaks, two at the start and one at the end of the gradient. Lysozyme activity was only present in the strongly bound proteins in the third peak (139 U/mL), and the inhibition profile made it possible to designate this activity as an i-type lysozyme (Figure 2A). Olsen et al. [18] performed large-scale isolation and enzymatic characterization of lysozymes from the blue mussel, leading to the identification of at least four distinct lysozymes, three from the style and one from the soft body. All four lysozymes were proposed to be of the i-type, based on a partial amino acid sequence and/or enzymatic properties, confirming findings from earlier studies that i-type is the dominant lysozyme in the blue mussel [17]. Protein blast analysis of *M. edulis* genomes in Genbank confirmed the presence of four gene alleles of putative i-type lysozymes (Supplementary Figure S3). 

### 2.3. Remaining Bacteriolytic Activity in Mussel Hemolymph Plasma after Lysozyme Removal by the Lys-Trap

The flow-through fractions collected after passage of mussel hemolymph plasma over the Lys-Trap array were found to still contain 69% of the initial bacteriolytic activity of the hemolymph plasma as measured by the *M. luteus* assay. Neither PliI, PliG, nor Ivy inhibited this activity, indicating that the Lys-Trap had effectively removed all the cognate i-, g-, and c-type lysozymes from the hemolymph plasma (data not shown). The bacteriolytic activity was caused by a proteinaceous compound since it was completely destroyed by pronase treatment (Supplementary Figure S4). The nature of this compound was not further studied, but there are at least two possible explanations. One possibility is that one or more lysozymes of the blue mussel are not recognized by the inhibitors used in our work, and hence passed through the Lys-Trap array. This seems most likely for c- and g-type lysozymes, considering that Ivy and PliG have been selected based on their inhibition of vertebrate c- and g-type lysozymes, respectively, and that these share only limited sequence similarity with their invertebrate homologs. PliI, on the other hand, was selected based on inhibition of the bivalve i-type lysozyme from *V. philippinarum* and is therefore closely related and thus likely to also recognize blue mussel i-type lysozymes. Alternatively, the lytic activity in the flow-through fractions could result from bacteriolytic hemolymph components different from lysozymes, such as proteases or peptidases that cleave peptidoglycan cross-links, bactericidal/permeability-increasing proteins (PBIs) or component-like proteins, all of which are present in bivalve hemolymph [17].

### 2.4. Role of i-Type Lysozyme and Its Bacterial Inhibitor PliI in the Survival of A. hydrophila in Mussel Hemolymph

*Aeromonas* are one of the few bacteria that encode inhibitors against the three major animal lysozyme types, and PliI was shown to contribute to i-type lysozyme tolerance in *A. hydrophila* upon outer membrane permeabilization with the natural compound lactoferrin [11]. Moreover, members of the *Aeromonas* genus are commonly associated with shellfish including bivalves [22], leading us to speculate whether PliI may be important for this relationship. Therefore, the survival of *A. hydrophila* wild-type, *A. hydrophila ΔpliI::aph* and *A. hydrophila ΔpliI::aph* (pFAJ1702-*pliI*) in mussel hemolymph was assessed. However, the viable cell numbers did not significantly change for any of the strains after 24 h in the hemolymph (Table 1). 

This means that, under the conditions of the experiment, PliI is not required for survival of *A. hydrophila* in mussel hemolymph. Since we previously demonstrated that *A. hydrophila* is sensitive to VpL only in the presence of an outer membrane permeabilizer like lactoferrin [11], it can be concluded that the mussel hemolymph is lacking factors that can permeabilize the *A. hydrophila* outer membrane, and that the bacteria were not phagocytosed by the hemocytes. 

### 2.5. Role of g-Type Lysozyme and Its Bacterial Inhibitor PliG on the Survival of E. coli in Mussel Hemolymph and Plasma

**Figure 3:**
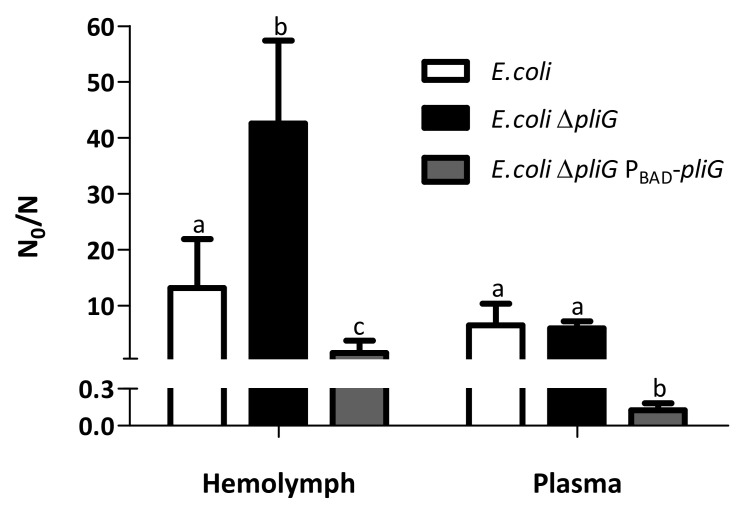
Survival *E. coli* strains in mussel hemolymph and plasma. Wild-type (white), *pliG* knockout (black), and complemented knockout (grey) *E. coli* strains were incubated for 24 h at 16 °C in blue mussel hemolymph or hemolymph plasma. Inactivation is expressed as the reduction factor N_0_/N, where N_0_ and N are the colony counts at the start of the experiment and after 24 h of incubation, respectively. Mean values + standard deviations from five independent experiments are shown. Significant differences (*p* < 0.05) between strains in each condition are indicated by different lowercase letters.

In addition to i-type lysozyme, g-type lysozyme was also detected in the hemolymph by the use of the Lys-Trap approach. Therefore, we also investigated the possible role of this lysozyme in antibacterial defense by analyzing the survival of *E. coli* wild-type, *E. coli* ∆*pliG*, and *E. coli* ∆*pliG* P_BAD_-*pliG* in mussel hemolymph in a similar way as for *A. hydrophila* (Figure 3). These strains were chosen because they were available from previous work in our lab and because *E. coli* is a frequent contaminant and possible health hazard of shellfish, particularly in fecally polluted estuarine waters [10,23]. The wild-type strain of *E. coli* was partially (13.2-fold) inactivated after 24 h. Deletion of PliG (∆*pliG*) significantly increased the inactivation (42.6-fold, *p* = 1.87 × 10^−3^), while overexpression of PliG (∆*pliG* P_BAD_-*pliG* strain) resulted in almost full survival (1.5-fold inactivation; *p* = 5.97 × 10^−4^). Since hemocytes have been shown to be an important reservoir of g-type lysozyme, we subsequently investigated the contribution of phagocytosis by mussel hemocytes to the observed inactivation by repeating the same experiment in hemolymph plasma. Removal of the hemocytes reduced the observed inactivation of all the strains, confirming that hemocytes contributed to bacterial clearance. The effect of PliG deletion on survival was no longer visible in the plasma (6.0-fold inactivation for wild-type and ∆*pliG*), but PliG overexpression actually supported growth (0.12-fold inactivation corresponds to 8.3-fold growth). Together, these results suggest that g-type lysozyme is present in the plasma and in the hemocytes and that PliG supports survival of *E. coli* in the hemolymph. Moreover, considering that *E. coli* does not have a *pliI* gene and its extracts lack inhibitory activity against the i-type lysozyme [11], the observation that overexpression of PliG allows it to grow in hemolymph implies that the i-type lysozyme is unable to inactivate *E. coli*.

## 3. Materials and Methods

### 3.1. Strains and Plasmids Used

The bacteria and plasmids used in this work are listed in Table 2. *E. coli* strains were grown in Luria–Bertani (LB) broth at 37 °C and *Aeromonas hydrophila* strains in nutrient broth (NB) at 30 °C. Antibiotics (Merck Life Science, Hoeilaart, Belgium) were added where appropriate at the following final concentrations: ampicillin (Ap), 100 µg/mL; kanamycin (km), 50 µg/mL; Tetracycline (Tc), 5 µg/mL.

### 3.2. Lysozymes and Determination of Lysozyme Enzymatic Activity

Hen egg white lysozyme (HEWL, c-type) was purchased from Merck Life Science. Salmon g-type lysozyme (SalG) and *Venerupis philippinarum* lysozyme (VpL) were produced recombinantly (see Table 2 for constructs) and purified as described elsewhere [11,24,25]. Lysozyme solutions were made in 0.1 M Tris-HCl pH 7.0 and protein concentrations were determined using a BCA Protein Assay Kit (Merck Life Science). The activity of the enzymes was determined with the *Micrococcus luteus* turbidity assay [8] using freeze-dried *M. luteus* (Merck Life Science).

### 3.3. Construction of Affinity Matrices with Immobilized Lysozyme Inhibitors

The lysozyme inhibitors Ivy, PliG, and PliI were expressed in *E. coli* (see Table 2 for constructs), extracted by cold osmotic shock and purified by Ni-affinity chromatography as described earlier [7,10,11]. Next, the inhibitor proteins were covalently coupled on 1 mL HiTrap^TM^ columns containing a N-hydroxysuccinimide (NHS)-activated chromatography resin for primary amine coupling (Merck Life Science), according to the instructions of the supplier. As such, three lysozyme inhibitor columns, designated c-Lys-Trap (with immobilized Ivy); g-Lys-Trap (with immobilized PliG), and i-Lys-Trap (with immobilized PliI), were constructed containing 9.2 mg, 5.5 mg, and 7.5 mg of effectively coupled inhibitor, respectively. 

### 3.4. Collection of Mussel Hemolymph

Blue mussels (*Mytilus edulis*) were purchased from a local retailer and hemolymph from individual animals was collected from the posterior adductor muscle using a needle and syringe, pooled and stored on ice. Hemolymph plasma was produced by removing the hemocytes from the hemolymph by centrifugation (6350× *g*, 10 min, 4 °C) and passage of the supernatant over a 0.22 µm filter. Hemolymph and plasma were stored at 4 °C until further use for maximally 24 h.

### 3.5. Lysozyme Profiling by Fast Protein Liquid Chromatography (FPLC)

The three affinity columns were coupled serially to an ÄKTA-FPLC^TM^ system in the order i-Lys-, g-Lys-, and c-Lys-Trap. Lysozyme profiling of mussel hemolymph comprised successive equilibration, sample loading and washing of the columns a flow rate of 1 mL/min in serial array, followed by elution of each individual column separately. Equilibration, loading, and washing was carried out in 0.1 M Tris-HCl pH 7.0, and a gradient of 0.1 M Tris-HCl pH 12.0 with 2 M KCl was applied for elution. 

### 3.6. Pronase Treatment 

Pronase (Merck Life Science) was used to verify whether the residual bacteriolytic activity detected in mussel hemolymph after passage over the serial lysozyme affinity trap can be ascribed to a protein. Fifty µL of a stock solution of pronase (20 mg/mL) in 10 mM of phosphate buffer pH 7.0 (and the same amount of buffer as a negative control) was added to 950 µL of lysozyme-free mussel hemolymph to obtain a final concentration of 1 mg/mL of pronase. After 24 h at 37 °C and the inactivation of pronase for 20 min at 80 °C, the lysozyme enzymatic activity in the samples was measured. 

### 3.7. In Vitro Survival of Bacteria in Mussel Hemolymph and Plasma

*E. coli* MG1655 wild-type, *E. coli* ∆*pliG*, and *E. coli* ∆*pliG* P_BAD_-*pliG* were grown overnight to stationary phase in LB broth with 0.1% arabinose to induce the P_BAD_ promotor. *A. hydrophila* wild-type (pFAJ1702), *A. hydrophila* Δ*pliI::aph* (pFAJ1702), and *A. hydrophila* Δ*pliI::aph* (pFAJ1702-*pliI*) were grown overnight to stationary phase in NB, diluted (1/100) in the same medium and grown to mid-exponential phase (OD_600nm_ = 0.6). Cells were washed three times with phosphate-buffered saline (PBS; 0.1 M KH_2_PO_4_, 0.1 M Na_2_HPO_4_, 0.15 M NaCl, pH 7.4). Fifty µL of bacterial suspension was then mixed with 450 µL of hemolymph or hemolymph plasma. Bacterial counts were determined immediately and after 24 h of incubation at 16 °C by plating appropriate dilutions on LB agar for *E. coli,* and on NB agar with Tc for *A. hydrophila*.

### 3.8. Bioinformatic Analysis

Homologs of the lysozymes used in this work (HEWL, SalG and VpL) in the bivalves *Mytilus edulis* and *Mytilus galloprovincialis* were retrieved from the Genbank non-redundant protein database by protein Blast analysis. Amino acid alignments were done using Clustal Omega [26].

## 4. Conclusions

This work reports the use of bacterial lysozyme inhibitors to detect, differentiate, and isolate the three major lysozyme types in animal body fluids or tissues. The method is rapid, relatively simple, does not require a priori knowledge of the studied animals, and complements the existing toolbox for the study of lysozymes as components of the innate immune system. The method allowed us to confirm the presence of the i-type lysozyme and demonstrate for the first time the presence of the g-type lysozyme in the hemolymph of the blue mussel. The results also suggested the absence of the c-type lysozyme, although genes encoding such lysozymes appear to be present. Further, challenge experiments of bacterial strains lacking or overexpressing specific lysozyme inhibitors in hemolymph allowed us to conclude that the g-type lysozyme plays an active role in the antibacterial immune function of the hemolymph. Although i-type lysozymes are generally considered to be more abundant and more important for immunity in bivalves, we failed to demonstrate any antibacterial activity by them against *A. hydrophila* and *E. coli*. These findings support the idea that the different types of lysozyme have complementary roles in controlling different groups of bacteria. Since the effectiveness of lysozymes also depends on other components such as outer membrane permeabilizers, our work highlights the complexity of the innate immune system of bivalves. The lysozyme profiling tool developed in this work can be expanded in the future with additional lysozyme inhibitors such as PliC/MliC [8], and could itself be used for the discovery of novel bacterial lysozyme inhibitors, by screening bacterial extracts for the presence of inhibitors against the compound(s) responsible for the residual bacteriolytic activity that we detected after the passage of hemolymph plasma over the Lys-Trap columns. 

## Figures and Tables

**Table 1:** Survival of *A. hydrophila* strains in mussel hemolymph at 16 °C.

Strain	Bacterial Count *	
	0 h	24 h
*A. hydrophila* (pFAJ1702) wild-type	2.3 ± 1.3 × 10^8^	1.5 ± 0.6 × 10^8^
*A. hydrophila* (pFAJ1702) Δ*pliI::aph*	1.5 ± 0.5 × 10^8^	1.5 ± 1.0 × 10^8^
*A. hydrophila* Δ*pliI::aph* pFAJ1702-*pliI*	1.9 ± 1.0 × 10^8^	1.1 ± 0.21 × 10^8^

* Mean values (CFU/mL) ± standard deviations from three independent replicate treatments. Changes in bacterial numbers after 24 h were not statistically significant (*p* < 0.05) for any of the strains.

**Table 2:** Strains and plasmids used.

Strain or Plasmid	Description	Reference or Source
*A. hydrophila* ATCC7966	Wild-type strain	American Type Culture Collection
*A. hydrophila* ∆*pliI*::aph	*pliI* gene replaced by *aph* gene; Km^R^	[11]
pFAJ1702-*pliI*	broad-host range vector pFAJ1702 carrying *pliI* with its own promotor; Tc^R^	[11]
*E. coli* MG1655	Wild-type strain	
*E. coli* ∆*pliG*	Markerless deletion of g-type lysozyme inhibitor gene *pliG* in *E. coli* MG1655	[10]
*E. coli* ∆*pliG* P_BAD_-*pliG*	*E. coli* ∆*pliG* with a chromosomal copy of *pliG* replacing the *araBAD* genes to bring *pliG* under control of the chromosomal arabinose-inducible P_BAD_ promotor	[10]
*Escherichia coli* BL21 (DE3)	Expression host for pET series vectors, containing IPTG inducible T7 RNA polymerase gene	Merck Life Science
*Escherichia coli* XL1-blue	Expression host, containing IPTG inducible T5 RNA polymerase gene	Agilent Technologies, Waldbronn, Germany
pET26b(+) (P_T7_-*pliI*)	*pliI* from *A. hydrophila* under control of P_T7_ promotor in pET26b(+); Km^R^	[11]
pET28b(+) (P_T7_-*pliG*)	*pliG* from *E. coli* under control of P_T7_ promotor in pET28b(+); Km^R^	[10]
pQE-0220 (P_T5_-*ivy*)	*ivy* from *E. coli* under control of P_T5_ promotor in pQE-60; Amp^R^	[7]
*Pichia pastoris* YJT46	Expression host for invertebrate lysozyme VpL of *Venerupis philippinarum* (*Tapes japonica*), methanol inducible	[24]
pQM64	Expression plasmid for g-type lysozyme SalG from the Atlantic salmon under control of P_T5_ promotor in pQE-02; Km^R^	[25]

## Data Availability

Data are contained within the article.

## Supplementary Materials

The following supporting information can be downloaded at: https://www.mdpi.com/article/10.3390/molecules28207071/s1. Figure S1: Amino acid sequence alignments and sequence identity percentages of hen egg white c-type lysozyme (HEWL) with its homolog(s) from *Mytilus edulis* and *Mytilus galloprovincialis*; Figure S2: Amino acid sequence alignments and sequence identity percentages of Salmon g-type lysozyme (SalG) with its homolog(s) from *Mytilus edulis* and *Mytilus galloprovincialis*; Figure S3: Amino acid sequence alignments and sequence identity percentages of *Venerupis (Tapes) philippinarum* i-type lysozyme (VpL) with its homolog(s) from *Mytilus edulis*; Figure S4: Pronase sensitivity of lysozyme activity present in complete hemolymph and in flow-through fraction after passage of hemolymph over the serial Lys-Trap.

## Sample Availability

Plasmid constructs are available upon request to the corresponding author.
