# Supplementary material for: Lysozyme Inhibitors as Tools for Lysozyme Profiling: Identification and Antibacterial Function of Lysozymes in the Hemolymph of the Blue Mussel
Source: Molecules. 2023 Oct 13;28(20):7071. doi: 10.3390/molecules28207071 (PMC10609593; doi:10.3390/molecules28207071)
Supplement: Supplementary file 1 [file molecules-28-07071-s001.zip › molecules-2546273-supplementary.pdf]

A

```
ACL81750.1      MRSLLILVLCFLPL----AA----LGKVFGRCELAAAMKRHGLDNYRGYSLGNWVCAAKF  52
CAG2215413.1    -------MHCSFPILLVLALVGYSYGATKTKCQVVQALRN---QNVPDSDLRDWLCLVKH  50
                       : * :*:    *      * .  :*::. *::.   :*  . .* :*:* .*.

ACL81750.1      ESNFNTQATNRNTDGSTDSGILQINSRWWCNDGRTPGSR-------NLCNIPCSALLSSD  105
CAG2215413.1    ESNYRYDVVGTNSNGSKDYGIFQMNSNYFCGRPSGTSSSTCWRVNTYGCADSCTSFTNSD  110
                ***:. :... *::**.* **:*:**.::*.      .*          *   *::: .**

ACL81750.1      ITASVNCAKKIVSDGNGMNAWVAWRNRCKGTDVQAWIRGCRL    147
CAG2215413.1    ISNDANCAVRVK-NCGGFGKWYGWRNHCSNVQGSEYDYSTC-    150
                *: ..*** ::  : .*:. * .***:*...: . :  .
```

	ACL81750.1	CAG2215413.1
ACL81750.1	100	28.89
CAG2215413.1	28.89	100

B

```
ACL81750.1      ---------MRSLLILVLCFLPLAALGKVFGRCELAAAMKRHGLDNYRGYSLGNWVCAAK    51
AJQ21500.1      MMDC-----NSVMVLFALLGCSY---AGTISKCDVVKALRAESV---PDSDLRDWLCLVE    49
VDI29468.1      --------------MRELASKRMRELASKRVSVQESARVSEQES---MSVSEYKRLCLVK    43
AJQ21538.1      -----MHFVCPILLVLALVGYSY---GATKTKCQVVQALRNQGV---PDSDLRNWLCLVK    49
AFM43653.1      MPSCGGILFVSILLVLVLVGYSY---GATKTKCQVVQALRNQGV---PDSDLRDWLCLVK    54
                              :  *        .      :    :  .      . .  . :* .:

ACL81750.1      FESNFNTQATNRNTDGSTDSGILQINSRWWCNDGRTPGS-------RNLCNIPCSALLSS    104
AJQ21500.1      HESSFRYELEHVNSDKSIDYGIFQLNNKYWCDRPTPVDTSKCWKFNTCGCTDTCTSLIDS    109
VDI29468.1      HESNYRYDVVGTNSNGSKDYGIFQMNSNYFCGRPSGTSSSTCWRVNTYGCADSCTSFTNS    103
AJQ21538.1      HESNYKYDVVGTNSNGSKDYGIFQMNSNYFCGRPSGTSTSTCWRVNTYGCADSCTSFTNS    109
AFM43653.1      HESNFHYDAIGTNS-GSKDYGIFQINSKFNCGRPSGTSTSICWRVNTYGCADSCTSLTNS    113
                .**.:. :    *:  * * **:*:*..: *.      .:           *   *::: .*

ACL81750.1      DITASVNCAKKIVSDGNGMNAWVAWRNRCKGTDVQAWI-RGCRL    147
AJQ21500.1      DISNDAKCAVQVK-NCNDFDEWYGWRDHCANVQSSEYDFSDC--    150
VDI29468.1      DISNDANCAVRVK-NCGGFGKWYGWRNHCSNVQGSEYDYSTC--    144
AJQ21538.1      DISNDANCAVRIK-KCGGFRKWYGWRNHCSNVQGAEYDYSTC--    150
AFM43653.1      DISNDAYCAVRIK-KCGGFSKWNGWKDYCSNVQGSEYDYSTC--    154
                **: .. ** ::  . ..:  * .*:: * ..:   :    *
```

	ACL81750.1	AJQ21500.1	VDI29468.1	AJQ21538.1	AFM43653.1
ACL81750.1	100	23.91	25.74	30.43	31.39
AJQ21500.1	23.91	100	47.52	56.85	58.39
VDI29468.1	25.74	47.52	100	73.05	63.57
AJQ21538.1	30.43	56.85	73.05	100	83.22
AFM43653.1	31.39	58.39	63.57	83.22	100

Figure S1. Amino acid sequence alignments (left) and sequence identity percentages (tables, right) of hen egg white c-type lysozyme (HEWL) with its homolog(s) from *Mytilus edulis* (A) *and Mytilus galloprovincialis* (B). Homologs were identified by protein Blast analysis using the HEWL sequence as a query against the Genbank non-redundant protein database. Sequences are named by their Genbank locus identifier. ACL81750.1 is HEWL. Alignment was done using Clustal Omega [26]. Identical residues are denoted by an asterisk (*), strongly conserved residues by a colon (:), and weakly conserved residues by a period (.).

A

```
ACI68650.1      ------------------------------------------------------------  0
CAG2231991.1    MFFLDAWFCVLKALQPRQHIHQDIAEINKHQSCYLQTGASNCIHPAVIAAVASRESRAGK  60
CAG2227921.1    ------------------------------------------------------------  0
CAG2231990.1    ------------------------------------------------------------  0

ACI68650.1      ---MGVGVGIGGF-ITFLTLASCFGDITKVDTSGASEITARQDKLTLQGVDASHKLAEHN  56
CAG2231991.1    LLYSTHGLGDKPQRLWYHADYNCHGDVTQLHPTGMGS--------AYGGMAGSHQAIDQD  112
CAG2227921.1    ------------MRNFYVA--DHNGDVTQLHPTGMVS--------AYGGVAGSCLAIDRD  38
CAG2231990.1    ------------MRNFYVA--DHNGDMTQLHPTGMVS--------AYGGVAGSCLAIDRD  38
                                : :  .  **:*::. :*  .        :  *: .*    :::

ACI68650.1      LVRMNKYKELITRVGQKHGLDPAIIAGIISRESRAGSAL--DHGWGDHGKSISVKALRFS  114
CAG2231991.1    IAEINKRKSCYVQAGAANCIHPAVIAGLASRESRAGKLLYSTGGWGDHHNAYGIMQCDIN  172
CAG2227921.1    IDELNKRKACYIQAGEKNGIPPAVIAAIASRESRAGRLLYKTNGWGDHHNAYGIMQCDIN  98
CAG2231990.1    IDELNKRKACYIQAGEKNGIPPAVIAAIASRESRAGRLLYKTNGWGDHHNAYGIMQCDIN  98
                : .:** *    :.*  : : **:**.: *******  *    ***** :: .:    :.

ACI68650.1      LSLSEGSRLNSLRGPRSTS-----------------------------------------  133
CAG2231991.1    AN-----PLHSIHKTCTSYHWDSCAHINAMTAHVLVPNIQGVKRKHHSWSDAQALQGGVA  227
CAG2227921.1    AN-----PLQSIYKTCTSYNWDSCDHINAMVEHVLSPNIQAVKRKHPSWSDAQALQGGVA  153
CAG2231990.1    AN-----PLQSIYKTCTSYNWDSCDHINAMVEHVLSPNIQAVKRKHPSWSDAQALQGGVA  153
                 .      *:*:  ::

ACI68650.1      ----------------------------------------     133
CAG2231991.1    AYNFGLGNVQSWGGLDVGSTHNDYSNDVIARAQWLISHYHW    268
CAG2227921.1    SYNFGVGNVQSWSGLDVGSTNDDYSNDVIARAQWLIRHYHW    194
CAG2231990.1    AYNFGVGNVQSWSGLDVGSTNNDYSNDVIARAQYLISHYHW    194
```

	ACI68650.1	CAG2231991.1	CAG2227921.1	CAG2231990.1
ACI68650.1	100	28.33	30.28	30.28
CAG2231991.1	28.33	100	79.38	79.90
CAG2227921.1	30.28	79.38	100	97.42
CAG2231990.1	30.28	79.90	97.42	100

B

```
ACI68650.1      -----MGVGVGIGGF-ITFLTLASCFGDITKVDTSGASEITARQDKLTLQGVDASHKLAE    54
AFQ35958.1      ----MENILVVLAVLVSVEAIDYNCHGNVTVLHPKGMA--------PKYGGMAASHLAID    48
OPL33468.1      ----MEMFLIVLAVLLSAEAINYNCHGNVTVLHPKGMA--------PKYGGMAASHLAID    48
VDI70518.1      MTTKMTSLHVHNG--LLVIISDYNCHGDVTQLHPTGMG--------SAYGGMAGSHQAID    50
AFQ35873.1      ----MKTFFLLSAVIFATDAANYNCHGDVTQLHPTGMG--------SAYGGMAGSHQAID    48
                       . :  .    .     .*.*::* :. .* .            *: .**   :

ACI68650.1      HNLVRMNKYKELITRVGQKHGLDPAIIAGIISRESRAGSAL--DHGWGDHGKSISVKALR    112
AFQ35958.1      RDITEMDKRKSCYLKAAANNCIHPAVIAGIASRESRAGKMLYSTNGWGDHHHAYGIMQCD    108
OPL33468.1      QDIAEINKRKSCYLKAAANNCIHPAVIAGIASRESRAGKMLYSTNGWGDHHHAYGIMQCD    108
VDI70518.1      QDIAEINKRKSCYVQAGAANCIHPAVIAGLASRESRAGKLLYSTSGWGDHHNAYGIMQCD    110
AFQ35873.1      QDIAEINKRKSCYVQAGAANCIHPAVIAGLASRESRAGKLLYSTSGWGDHHNACGIMQCD    108
                :::..::* *.   :..  : :.**:***: *******. *    ***** :: .:

ACI68650.1      FSLSLSEG------------SRLNSLR---------GPRSTS-----------------    133
AFQ35958.1      VRVDPLHPYNKNCTSFLWYSCDHINAMTKYVLVPYIEAVKQKLPSWSDAQALQGGVAAYN    168
OPL33468.1      VRVDPLHPYHKNCTSYPWYSCDHINAMIKYVLVPYIEAVKQKLPSWSDAQALQGGVAAYN    168
VDI70518.1      INANPLHSIHKTCTSYHWDSCAHINAMTAHVLVPNIQGVKRKHHSWSDAQALQGGVAAYN    170
AFQ35873.1      INANPLHSIHKTCTSYHWDSCAHINAMTAHVLVPNIQGVKRKHHSWSDAQALQGGVAAYN    168
                .  .  .               ::*::          . : .

ACI68650.1      --------------------------------------  133
AFQ35958.1      FGVRNVRTWDKLDIGTTHNDYSNDVIAQAQWLIHRYNW  206
OPL33468.1      FGVRNVRTWDKLDIGTTHNDYSNDVIAQAQWLINRYKW  206
VDI70518.1      FGLGNVQSWGGLDVGSTHNDYSNDVIARAQWLISHYHW  208
AFQ35873.1      FGLGNVQSWGGLDVGSTHNDYSNDVIARAQWLISHYHW  206
```

	ACI68650.1	AFQ35958.1	OPL33468.1	VDI70518.1	AFQ35873
ACI68650.1	100	25.60	24.80	27.64	26.40
AFQ35958	25.60	100	92.23	68.63	67.48
OPL33468.1	24.80	92.23	100	70.10	70.87
VDI70518.1	27.64	68.63	70.10	100	92.16
AFQ35873	26.40	67.48	70.87	92.16	100

Figure S2. Amino acid sequence alignments (left) and sequence identity percentages (tables, right) of Salmon g-type lysozyme (SalG) with its homolog(s) from *Mytilus edulis* (A) *and Mytilus galloprovincialis* (B). Homologs were identified by protein Blast analysis using the SalG sequence as a query against the Genbank non-redundant protein database. Sequences are named by their Genbank locus identifier. ACI68650.1 is SalG. Alignment was done using Clustal Omega [26]. Identical residues are denoted by an asterisk (*), strongly conserved residues by a colon (:), and weakly conserved residues by a period (.).

```
AMS37097.1        ---------------------------------------------MLGVF--------  5
CAG2239847.1      MRFDKTRRNTSNVQTQAQLYL-----KKTCCGHLDTVPFTVQSTYGEMYVLFVTDNTVAR  55
ABB76765.1        ------------------------------------------------------------  0
AAN16207.1        --------MMTELKMSVALFFALLCGLNVCCGLKEIL-----------------------  29
CAG2221082.1      --------------MSVALFFALLCGLNVCCGLKEIL-----------------------  23

AMS37097.1        ---VFSVLL----------NMAMSGVIENPVSLMCLNCMCKVATTSCTDVGCNHDREGIA  52
CAG2239847.1      EGFRISYILEGAP--NTTARTNHDREDSKLPMYLAVGCAILVESNCDKTIGCHDDGGSDS  113
ABB76765.1        -------------------------ATGVVSDKCMQCICDLESGCR-PLDCRWDVNSNS  33
AAN16207.1        ESYKVEFEQREVDVESDEPASDDSIDSNGLVSDKCMRCICMVESHCNNNIGCRMDVGSLS  89
CAG2221082.1      ESYKVEFEQREVDVESDEPASDDSIDSNGLVSDKCMRCICMVESHCNNNIGCRMDVGSLS  83
                                                  .: *   : : .   :.*. *  . :

AMS37097.1        CGYYQIHYGYYIDCYKPVPDSGCRGLSEQECWMKCSRDKPCAERCIQSYMLRYRNKNGAG  112
CAG2239847.1      CGPFQIKEVYWIDCNRPGDS-----------YEECTKDYKCAKGCVQAYMERYGG-WKCG  161
ABB76765.1        CGYMQIKQVYWDGCGKPGGS-----------LEACSKDKHCASQCVQKYMFRYINHYGCA  82
AAN16207.1        CGPFQIKKAYWIDCGQPKGD-----------YKTCANDYSCAYNCIQTYMARYIGHSGCP  138
CAG2221082.1      CGPFQIKKAYWIDCGQPKGD-----------YKTCANDYSCAYNCIQTYMARYIGYSGCP  132
                  **  **:  *: .* :*  .              *:.*  **  *:* ** **  .   .

AMS37097.1        SDCENYVRLHAGGPYGAAKWNQSWYNRTSYYWNYARAYGCVWNS  156
CAG2239847.1      TTCEDYARMHNGGPKGYKKSA------TDVYWNKIRNAGCSSDS  199
ABB76765.1        HNCESYARMPNGGPAGCKHIN------TLGYGSHIQSKGCSANS  120
AAN16207.1        KNCESYARIHNGGPRGCTNPN------TIGYWNKIKQQGCTIYS  176
CAG2221082.1      KNCESYARIHNGGPRGCTNPN------TIGYWNKIKQQGCTIYS  170
                    **.*.*:  *** *  :        *  * .  :  **   *
```

	AMS37097.1	CAG2239847.1	ABBB76765.1	AAN16207.1	CAG2221082.1
AMS37097.1	100	34.06	37.50	36.57	36.57
CAG2239847.1	34.06	100	44.54	38.69	40.12
ABBB76765.1	37.50	44.54	100	54.17	53.33
ABBB76765.1	36.57	38.69	54.17	100	99.41
CAG2221082.1	36.57	40.12	53.33	99.41	100

Figure S3. Amino acid sequence alignment (left) and sequence identity percentages (tables, right) of *Venerupis (Tapes) philippinarum* i-type lysozyme (VpL) with its homolog(s) from *Mytilus edulis*. Homologs were identified by protein Blast analysis using the VpL sequence as a query against the Genbank non-redundant protein database. Sequences are named by their Genbank locus identifier. AMS37097 is VpL. Alignment was done using Clustal Omega [26]. Identical residues are denoted by an asterisk (*), strongly conserved residues by a colon (:), and weakly conserved residues by a period (.).

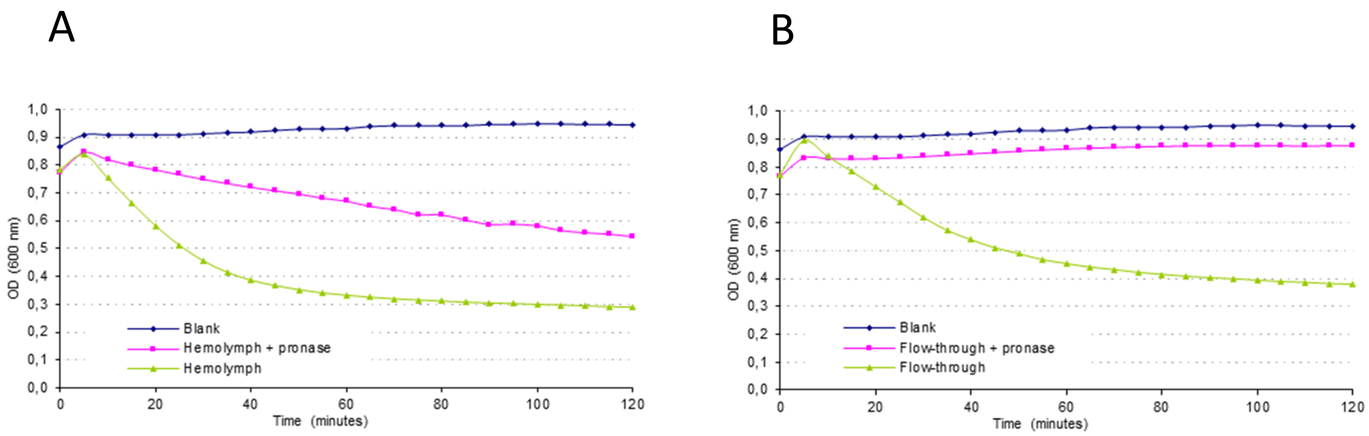

Figure S4. Pronase sensitivity of lysozyme activity present in complete hemolymph (A) and in flow-through fraction after passage of hemolymph over the serial Lys-Trap (B). Lysozyme activity was measure using the *Micrococcus luteus* turbidity assay. Blank is a *M. luteus* suspension with pronase but with water replacing the test sample.

Reference

26 Sievers, F.; Wilm, A.; Dineen, D.; Gibson, T.J.; Karplus, K.; Li, W.; Lopez, R.; McWilliam, H.; Remmert, M.; Söding, J.; Thompson, J.D.; Higgins, D.G. Fast, scalable generation of high-quality protein multiple sequence alignments using Clustal Omega. *Mol. Syst. Biol.* **2011**, *7*, 539. https://doi.org/10.1038/msb.2011.75.
